# Parents’ Perceptions of the Effectiveness of Music Therapy on Their Chronically Ill Children

**DOI:** 10.3390/bs13050409

**Published:** 2023-05-14

**Authors:** Susann Kobus, Alexandra M. Buehne, Simone Kathemann, Anja K. Buescher, Elke Lainka

**Affiliations:** 1 Center of Artistic Therapy, University Medicine Essen, 45147 Essen, Germany; 2 Clinic for Pediatrics I, University Children’s Hospital Essen, University of Duisburg-Essen, 45147 Essen, Germany; 3 Clinic for Pediatrics II, University Children’s Hospital Essen, University of Duisburg-Essen, 45147 Essen, Germany

**Keywords:** music therapy, children, pediatrics, chronic diseases, parents’ perception

## Abstract

Chronic disease in a child, with the associated hospital stays, places considerable demands on the child and their family. The aim of this study was to investigate the parents’ perceptions of the music therapy used with their child during a hospital stay and to determine whether they felt that it reduced the child’s anxiety and stress generated by hospital admission. We hypothesized that the use of live music therapy from a music therapist would positively support these patients in everyday clinical practice, promote their wellbeing, and have positive impacts on their vital signs and blood pressure. Children with chronic gastroenterological and nephrological diseases included in this prospective study received live music therapy with a median duration of 41 min (range from 12 to 70 min) two to four times per week until discharged from the hospital. At the time of discharge, the parents were asked to complete a Likert-style questionnaire to evaluate the music therapy. Seven items were related to general questions about the patients and sessions, and eleven items evaluated the personal perceptions of the parents. Music therapy was conducted in 83 children, with a median age of 3 years (range from 1 month to 18 years). All parents (100%) completed the questionnaire at the time of discharge. Seventy-nine percent of the parents stated that their children were able to enjoy the music therapy sessions without being stressed. In addition, 98% of the respondents said that they were grateful for the music therapy their children received (97% fully agreed and 1% rather agreed). All parents considered music therapy to be beneficial for their child. The parents’ responses reflected the view that music therapy is beneficial to patients. According to the parents, music therapy can be integrated effectively in the inpatient clinical setting and can support children with chronic illnesses during their hospital stay.

## 1. Introduction

The primary burden of a disease in children and adolescents up to the age of 18 has increased enormously in recent years. Mild infectious diseases have developed into chronic diseases [1,2,3]. The frequency of chronic diseases in children worldwide has risen sharply [2]. While the chances of survival for preterm infants [4] and children with diseases [5] has improved, children and adolescents have more mental, behavioral and developmental disorders today [6]. The care of children and infants with chronic diseases in medicine has improved enormously, meaning that diseases that used to be fatal are medically treatable today [7,8]. Depending on the definition used, 13–27% of children suffer from a chronic disease [2]. The prevalence of chronic diseases in children and adolescents aged 0 to 17 in Germany is 16.2% [9].

Chronic disease affects many aspects of children’s lives, with consequences that continue into adulthood. For this reason, the high prevalence of such diseases is a key public health challenge [10], and hospital care plays an important role in diagnosis and treatment [11]. The aim of a considerable portion of care in chronic childhood illness is to improve quality of life [12].

About one third of girls and one fifth of boys suffer from multiple psychosomatic complaints in Germany [13,14]. Impairments in subjective well-being manifest themselves mainly in girls, older adolescents, and young people with less family prosperity and high levels of school stress. Strong family support is associated with better subjective well-being [13]. A hospital stay means an additional stress factor for chronically ill children and adolescents [15,16]. Meaningful engagement with pediatric patients in projects can support the patients’ development during their hospital stay [17]. The presence of a parent; interventions such as art therapy, music therapy, sports therapy, and physiotherapy; and visits from the psychosocial service, the hospital school, and clinic clowns can promote psychological health during hospital treatment [18,19,20,21]. Music therapy, a clinical and evidence-based use of music interventions to accomplish individualized aims within a therapeutic relationship [22], can be used to promote children’s and parents’ psychological wellbeing during their hospitalization [23]. This kind of non-drug intervention is effective in reducing pain and anxiety [23,24,25,26]. Live music therapy has a stabilizing effect on the vital signs of heart rate, oxygen saturation [27], and respiratory rate and blood pressure [28]. It is effective regardless of the wellbeing of the patient and usable at intensive care units (ICUs). The intensity of pain can be reduced, and pain tolerance can be increased [29]. Music therapy interventions can also improve communication, promote physical rehabilitation, and enhance memory and the expression of feelings [22]. A growing interest in music therapy improves its integration and further development in basic medical care interventions [30]. A study of patients on a cardiovascular unit who were interviewed individually about the live music they had experienced showed that music therapy provides comfort and relaxation for patients during their hospital stay [31]. In a study with preterm infants, live performed music therapy was perceived by parents as a positive, supportive offering for their children. Music therapy care offered helpful relief to the families of preterm infants during the difficult time of their hospital stay [32].

Based on the previous findings with preterm infants [27,32] and research results in the fields of family-centered nursing care and family-centered music therapy in the inpatient setting [22,29], we investigated how the parents of chronically ill children and teenagers perceived the music therapy care their children received during hospitalization, regardless of whether the children were treated in an ICU or on a pediatric special care unit (SCU). We hypothesized that live music therapy performed by a music therapist, where music is listened to and improvised both on instruments and vocally, supports the patients in everyday clinical practice and promotes parents’ perceptions of the wellbeing of their child.

## 2. Methods

### 2.1. Study Design

The results of the current survey were collected as part of a prospective clinical study on music therapy in hospitalized children with chronic gastroenterological and nephrological diseases [28]. The results were collected anonymously as a blinded study. The parents of these hospitalized children with chronic gastroenterological and nephrological diseases were given the questionnaires evaluated in this additional part of the study presented here to fill out before their children were discharged. Depending on their medical status, the children were treated in a pediatric ICU or SCU and received the appropriate type of music therapy, either active or receptive, from a qualified music therapist. In active music therapy, the patient played improvised or composed melodies on instruments, either alone or with the music therapist. No previous musical knowledge was required for this. In receptive music therapy, the music therapist played improvised or composed melodies and the patients listened to the music. The music therapist informed the patients and parents of the study. The declarations of consent were signed by the parents and, in the case of patients over the age of 7, by themselves and their parents. The Likert-style questionnaire was completed by the parents. 

### 2.2. Setting and Procedure

The duration of a music therapy session was variable depending on the patient’s health status. The study was conducted in the nephrological and gastroenterological wards in the University Children’s Hospital Essen. Recruitment and information were provided by the senior physician. The local ethics committee of the Medical Faculty of the University of Duisburg-Essen approved the main and additional parts of the study presented here, both of which are registered in the German Clinical Trials Register (DRKS00026158).

### 2.3. Eligibility and Recruitment

All parents of children up to the age of 18 with chronic gastroenterological and nephrological diseases who were treated in the University Children’s Hospital Essen between November 2020 and October 2021 and received music therapy care could take part in the study. It made no difference for our study whether the patients received medical care in the ICU or the SCU because music therapy was performed with or for the children regardless of their health status. Reasons for exclusion included insufficient German language skills to understand the study goals, a lack of interest in participating in the study on the part of the children and parents, and a too-short hospital stay. Patients who were admitted for diagnostic purposes and where it was known that they would be treated as inpatients for fewer than five days did not receive music therapy. Therefore, they were not included in the study. Further reasons for exclusion included difficulties in organizing the therapy because of time constraints around many clinical examinations, and palliative care patients receiving music therapy in this setting.

### 2.4. Sample Size and Sampling Methods

The calculation of the sample size with a population size of 70 patients, a margin error of 5% and a 95% confidence level indicated that at least 60 patients should be included. We used the probability sampling method in the form of a simple random sampling. The children had to be chronically ill, in medical care at the pediatric nephrology or gastroenterology wards, and under 18 years of age. Before the inclusion into the study, they were not allowed to receive any music therapy sessions in the University Children’s Hospital Essen.

### 2.5. Intervention

Clinically stable children up to the age of 18 years received active or receptive music therapy two to four times a week from admission until discharge. The timing was variable depending on the clinical situation. The intervention program aimed to promote the well-being of the patients and their parents and to give positive support to these patients and parents in everyday clinical practice. Each music therapy intervention was performed individually by one of the two available qualified music therapists in the patient’s room directly at their bedside. Different instruments including several percussion instruments and therapeutic instruments such as sansula, kalimba, wah-wah tubes, ocean disc and zaphir, xylophones, tone bars, egg-shakers, vibraslaps, cajons, bongos, djembes, a keyboard, and a guitar were used, and no prior musical knowledge was required from the patients [33]. If the children were too weak or tired and unable to play music actively, the patients could express their wish to switch the active music therapy treatment to receptive music therapy. In receptive music therapy, the music therapist usually played a sansula, some chimes, an ocean disc, or a zaphir. In both types of interventions, the patient decided the content of the sessions. The therapist and the patient improvised freely or individually composed music or songs. In other cases, children’s songs or pop songs were sung. The patients were free to decide which musical directions they preferred at the moment. There were no differences in approach between children younger than 1 year and those older than 1 year. The music therapist decided how to treat each patient individually, depending on their motoric, cognitive, and physical abilities. When the parents were present during an intervention, they were only in the therapy room as observers. After the last music therapy session before discharge, questionnaires were filled out by the observing parents who were not actively involved in the music therapy. Since for developmental reasons not all children could read and write, the parents’ perceptions were used for better comparability.

### 2.6. Questionnaires

At the time of discharge, parents filled out an anonymous Likert-style questionnaire of their perception of the music therapy treatment during hospitalization. The Likert scale as a bipolar rating scale was well suited for this study because it can capture moods based on individual questions. The questionnaire was developed by the authors based on a questionnaire used in a study with preterm infants born before 32 weeks of gestation [32]. Seventy-four mothers and nine fathers filled out the questionnaire. The parents indicated how their child felt during and after the therapy and how they perceived their child during the therapy in which the parents were present. The observations of the parents were related to the therapy units where the parents were present. The questionnaire consisted of two parts. There were seven items relating to general questions about the age and the illness of the patient, the treating ward, the kind of music therapy performed, their mother language, and who had filled out the questionnaire. Nominal scales were used for these items. Eleven additional items evaluated the personal perceptions of the parents about the music therapy care and the patients’ feelings using an ordinal scale for eight items and nominal scales for three more items. For the eight items with the ordinal scale, we used a five-point scale counting the number of respondents and asked whether the music therapy support represented a positive change and enrichment during the clinical stay, whether the child was able to relax well without having to exert themself, whether the child was able to repress thoughts about their illness to some extent during the music therapy, whether the child had gained more self-confidence from the therapy, whether the child had developed an enjoyment of singing during the course of the therapy, whether the therapist was empathetic and understanding, if there had been anything that had disrupted the music therapy sessions, and if the parents were grateful for the music therapy their child received during their clinical stay. In the three questions with nominal scales, we asked about the parents’ perceptions of the condition of their child or adolescent during and after the music therapy sessions and whether the music therapy was good for them.

The survey has already been used in a modified form and corresponds to the specifications of the cited analysis on rating scales [34]. The questionnaire was handed out to the parents by the music therapist and completed anonymously. On completion, the parents put the questionnaire in an envelope into the mailbox of the music therapist. The parents knew that the mailbox belonged to the music therapist for study purposes. No conclusions could be drawn about which questionnaire belonged to which patient because several patients took part in the study at the same time and the mailbox was not emptied until the end of the survey.

### 2.7. Bias

To preserve the blinding, the parents put the completed questionnaire anonymously in the music therapist’s mailbox so that no tracing of who filled out the questionnaire could be made. The music therapy process of each child was documented by the qualified music therapist in a standardized form. To maintain blinding, no notes of the music therapy sessions were entered into the medical record of the patients.

### 2.8. Statistical Analysis

The characteristics of the patients and the music therapy interventions were given as absolute frequencies and percentages. Descriptive analyses included median and range for non-normal distribution, as well as absolute and relative frequencies for categorical data. The statistical analysis of the individual Likert items of the written survey is based on absolute and percentage frequencies. Anonymized data and all graphics were analyzed and created using Microsoft 365 Excel. All statistical calculations were performed using IBM SPSS Statistics 27 (IBM, Chicago, IL, USA). *p*-values < 0.05 were considered significant.

## 3. Results

### 3.1. Patients

**Figure 1:**
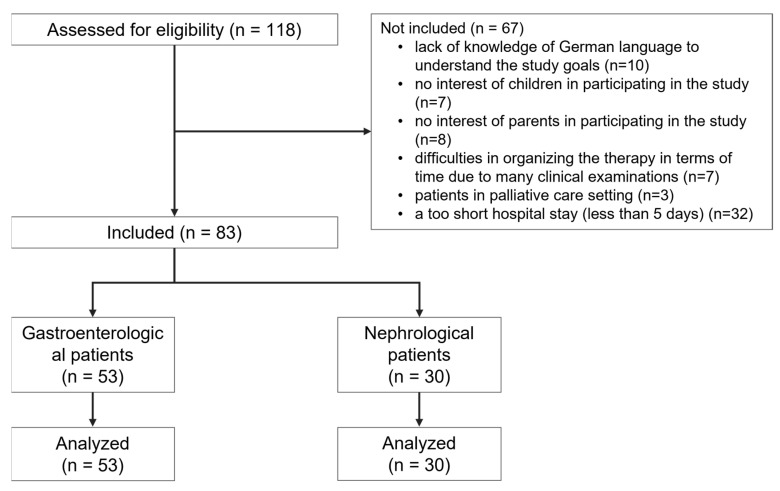
Flowchart of the included and excluded participants of the study.

Overall, 83 children (male 48, female 35) who were treated at the gastroenterological (n = 53) and nephrological (n = 30) wards at the Clinic for Pediatrics II at University Hospital Essen, Germany, were included in the study (Figure 1). 

Those included had a median age of 3 years (gastroenterological patients: 5 years, nephrological patients: 2 years) at the first therapy session. Thirty patients were less than 1 year old. A total of 45 patients had German, and 38 patients spoke other languages, such as Albanian (7), Arabic (8), French (1), Greek (2), Italian (2), Moroccan (1), Pakistani (1), Persian (1), Polish (1), Russian (2), Serbian (1), Spanish (2), Czech (2), and Turkish (7) as their native tongue. The average length of hospital stay of the recruited patients was 22 days (range from 7 to 169 days). All children had a primary diagnosis of liver or kidney disease. On average, the patients spent a fifth of their hospital stay in the ICU. The characteristics of the patients and the music therapy sessions are shown in Table 1.

### 3.2. Music Therapy Sessions

In total, 377 music therapy sessions were conducted with children and adolescents up to the age of 18 years. Of these, 298 (79%) therapies were carried out in the SCU and 79 (21%) therapies were carried out in the ICU. Parents were present as observers in 220 (58%) sessions, but they were not involved during the therapy. The median duration of each music therapy session was 41 min (range from 12 to 70 min). For 47 children, the parents were present at every music therapy session. Every child was accompanied by a parent. On average, the parents were present in 74% of their child’s music therapy sessions. A total of 39% of the patients received receptive music therapy exclusively, whilst 29% received active music therapy alone. In 23% of the participating children, receptive and active music therapy sessions were conducted in equal proportions. Active music therapy dominated in 7%, and receptive music therapy dominated in 2%. When analyzing the age groups, it becomes apparent that babies under the age of 1 only listened to music. The number of active music therapy sessions increased with increased age (Table 2). 

### 3.3. Questionnaires

The parents completed the questionnaires after their last therapy session before discharge. This last therapy session was either on the day of discharge or the day before, when it was already confirmed that the child would be discharged the next day. We received completed questionnaires from all 83 patients. The mothers of 74 patients (89%) and the fathers of 9 patients (11%) completed the questionnaires. Independently of whether the mother or the father was present during the therapy sessions, most of the interventions were receptive music therapy (39%) (Table 2).

The answers to the questionnaires showed that the parents perceived the music therapy support positively (Table 3). All parents stated that the music therapy support offered an opportunity for positive change and enrichment during the clinical stay, and 98% of the respondents said that they were grateful for the music therapy that their children received. A total of 95.2% of the parents stated that their children were able to relax without having to exert themselves, and 80.7% of the children were able to repress thoughts of their illness to a certain extent during music therapy. The remaining 19.3% of parents indicated that they could not judge this question. All these parents had children under the age of 4 years (37% of the children were under 1 year and 63% were 1 to 3 years old). Furthermore, 65.0% of the parents indicated that the child gained more self-confidence during the therapy, but 30.2% also could not answer this question (24% of these children were aged less than 1 year, 68% were aged 1 to 3 years, and 8% were 11 to 14 years old). In addition, 64.9% had developed an enjoyment of singing during the therapy. Some parents (4.8%) said that there were things which had disturbed the music therapy sessions. They complained that there was too much noise throughout the unit due to other children crying, sounds in the corridor facing the patient’s room, and noise from devices and machines and sometimes from construction work. All parents were satisfied with the music therapist, considered that the music therapist felt empathy with their child, and noticed that the children felt understood. 

**Figure 2:**
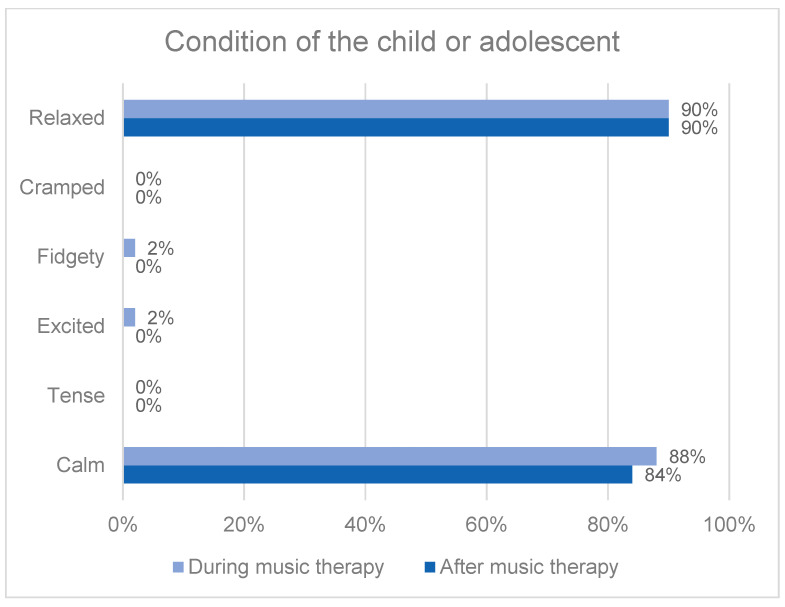
Parents’ perception of the condition of their child or adolescent during and after a music therapy session.

During music therapy, 88% (n = 73) of the parents rated their children as calm, and 84% (n = 70) of parents considered that this continued even after music therapy. Likewise, 90% (n = 75) of the children looked relaxed to their parents during music therapy, and 90% (n = 73) looked relaxed even after music therapy (Figure 2). In our study, 2% (n = 2) of the parents observed that their child felt excited during the music therapy, and 2% (n = 2) were able to see this even after the music therapy session was over. Overall, all parents stated that music therapy was good for their children.

### 3.4. Parents’ Perceptions with Different Inpatient Statuses

In the case of 53 children (64%), the parents were admitted as inpatient companions because their children were either too young or needed extensive help (not because of needing treatment themselves). The mean age of the children whose parents were also hospitalized was 3.5 years (range from 1 month to 13 years) and that of the children whose parents were not hospitalized was 2 years and 8 months (range from 1 month to 17 years). The parents of 28 patients (34%) were not hospitalized. Regarding 17 parents, the children were in the ICU, where parents were not allowed to stay the night. For three children, the parents could not live in the clinic for family reasons, and the eight children who had an average age of 15.5 years were old enough to stay in the clinic alone. Two more children (3%) changed wards from an ICU to an SCU. In the ICU, the parents were not hospitalized but they were admitted as inpatients when the children were transferred to the SCU.

Regardless of the carers’ different inpatient statuses, all parents stated that music therapy represented a positive change and enrichment during the clinical stay (both inpatient statuses: 96.4% fully agreed and 3.6% rather agreed). All parents noticed that their children felt understood by the empathetic music therapist. When asked whether the children were able to repress thoughts of their illness during music therapy, 83.0% of the inpatient parents fully agreed and 46.4% of the non-inpatient parents fully agreed. Regarding self-confidence, 66.0% of inpatient parents fully agreed that their children gained more self-confidence during therapy, compared with 46.4% of the non-inpatient parents (Table 3).

## 4. Discussion

The aim of this study was to examine parents’ perceptions of the music therapy given to their inpatient child. All parents reported that the music therapy support offered positive change and enriched their child’s clinical stay. 

The results of our study showed that nearly all parents were generally satisfied with their child’s music therapy program. Parents responded that their child felt relaxed and calm within the musical environment. This accords with various studies on the perception of music therapy, which have shown that patients with different clinical pictures and healthcare professionals attributed positive effects to music therapy care [29,30]. Parents reported that music therapy made their children calmer and more relaxed [35]. 

Our results were also in line with the perceptions of parents whose preterm infants received live music therapy from a qualified music therapist who involved the parents during their hospital stay from one week after birth until discharge. Music therapy was perceived as a positive, supportive offer both for themselves and for their infants. The therapy promoted relaxation in parents and infants as well as in parent–child interactions. Music therapy helps to support the families of prematurely born infants in the difficult early days of life [32].

The results of this study indicate that music therapy can be a viable psychosocial intervention for patients with chronic diseases. Therefore, we can confirm the effectiveness and stabilizing effect of music therapy, which has already been shown in the improved vital signs of heart rate and oxygen saturation after music therapy interventions [28]. 

The results of our current study complement findings that music therapy is perceived positively and promotes relaxation in patients during their stay in the clinic [36]. Music therapy was associated with positive perceptions of support by all our parents. There was a significant amount of gratitude among patients who received music therapy care [32], and 98% of the parents of the participants in our study were also grateful for the music therapy they received. The missing 2% of those who filled out the questionnaires stated “I don’t know”. Music therapy supports patients during hospitalization until their discharge. This was also demonstrated by the results of a study with psychiatric patients, who rated music therapy as significantly more helpful than all other programs. Participants who were admitted to a psychiatric institution consistently rated music therapy as more effective than any other type of therapy addressing specific areas of psychiatric difficulty [37].

Overall, 20% of the parents could not make a judgement about the question of whether their children were able to suppress thoughts of their illness to a certain extent during music therapy; 37% of the children in question were under 1 year old, and 63% were aged 1 to 3 years. In addition, 30% of the parents could not say whether their child gained more self-confidence during the therapy (24% of the children were under 1 year old, 68% were aged 1 to 3 years, and 8% were aged 11 to 14 years). From these results, we can conclude that the children and adolescents themselves should be questioned on the topics of self-confidence and their thoughts about their illness in a further study. However, clearly these are not topics for babies and toddlers and only the older children would be able to comment.

When analyzing the parents according to their inpatient status, we saw that both groups of parents observed that music therapy effected positive change and enrichment during the clinical stay. All parents were grateful for the music therapy support regardless of whether their child was hospitalized. More inpatient parents saw that their children were able to repress thoughts of their illness during the music therapy sessions (83% inpatient parents and 46% non-inpatient parents), and more inpatient parents saw an improvement in their children’s self-confidence compared with the non-inpatient parents (66% inpatient parents and 46% non-inpatient parents). The parents admitted with their children could observe their children for whole days and nights compared with the parents who returned home from time to time. Overall, 46% of the non-inpatient parents compared with 23% of the inpatient parents could not assess whether their child had gained more self-confidence in therapy. Some parents were at the children’s hospital for only a very short time each day, whilst other parents stayed for several hours a day. Music therapy could have an influence on sleep quality, but this could only be observed by inpatient parents [38]. More research is needed to show whether music therapy also increases self-confidence in children where the parents are not present at the music therapy intervention. Children and adolescents are more relaxed and uninhibited when their parents are not around [39]. In a further study, test methods will be used to investigate the effects of music therapy care on self-confidence and other issues, to be assessed specifically by the patient from school age until adulthood.

Music therapy care has the advantage of creating an additional activity to offer a distraction from everyday hospital life and illness [40,41,42]. Music therapy can distract from the stressful hospital environment, and in a supportive interaction with the therapist, the child can discover personal resources [43]. It creates an environment in which elements of everyday life are present and contributes to making a hospital space less foreign and hostile [44]. The children have fun, and time flies faster than when they sit or lie alone in a hospital bed [45]. In receptive music therapy, they can find relaxation, inducing an observable state of calm. This is promoted in both receptive and active music therapy [46]. A general improvement in mood is visible. Loneliness, anxiety, and agitation are also reduced [32,47]. Music therapy improves children’s abilities to cope with challenges encountered in the hospital setting, including wellbeing anxiety, boredom, and loss of control. The music evokes memories and connects patients to their past. It provides a human connection and emotional support through dialogue, song, and listening [32]. In addition to clinical examinations and interventions, there is a positive aspect to daily routine care in which the children are free from the medical regimen [28]. The anticipation of the music therapy and increased motivation within music therapy can have an overall positive effect on communication with the child [48].

Our study has some limitations. To avoid confounding and disparities inherent in the broad sampling of ages, we only surveyed the parents. Apart from the babies, there were also children who were not able to read on their own or who could not understand the questionnaires. In a further study, surveys should also be carried out by the patients themselves. There was no comparison with a control group who received no music therapy intervention or received a different type of therapy. The validity of our Likert-style questionnaire was not tested on a randomized study. The limitations of our analysis include the heterogeneity of patients and different disease groups. Recruitment bias may have influenced the results, as children or parents who were not interested in music therapy were not included in the study. A future study is needed to explore whether music therapy influences the quality of life of hospitalized, chronically ill children. In an additional study, the perceptions of the parents will be compared with the perceptions of the children and young people themselves. The Assessment of Parent–Child Interaction Manual (ACPI), a tool in the field of family-centered music therapy, will also be used for these analyses.

## 5. Conclusions

We presented the results of a survey aimed at understanding parents’ perceptions and experiences of music therapy for their children. Through the survey, we found that all parents considered the music therapy to enrich and positively affect their children during the everyday hospital experience, and nearly all parents stated that they were grateful for the music therapy. Music therapy should be further expanded in medical care, regardless of the medical specialty. Due to the effectiveness of music therapy as part of medical care in the clinical setting, it would be appropriate for German health insurance companies to cover these costs.

## Figures and Tables

**Table 1:** Characteristics of participants and music therapy sessions.

	Gastroenterological Patients	Nephrological Patients	Alle Patients
Patients, n (%)	53 (64)	30 (36)	83 (100)
Native language German Other languages	26 27	19 11	45 38
Number of music therapy sessions, n (%) active music therapy receptive music therapy music therapy at ICU music therapy at SCU	228 (60) 67 (38) 161 (81) 73 155	149 (40) 110 (62) 39 (19) 6 143	377 (100) 177 (100) 200 (100) 79 298
Median duration of music therapy sessions, min. (range) active music therapy receptive music therapy music therapy at ICU music therapy at SCU	38 (12–60) 47 (20–60) 35 (15–70) 33 (18–60) 41 (15–70)	45 (15–70) 48 (20–60) 36 (12–60) 28 (20–35) 45 (12–60)	41 (12–70) 47 (20–60) 35 (12–70) 32 (18–60) 41 (15–70)
Median age, years at music therapy	5	2	3

ICU: intensive care unit; SCU: special care unit.

**Table 2:** Characteristics of the performed music therapies in relation to age groups of patients.

	Exclusively Receptive Music Therapy, n (%)	Exclusively Active Music Therapy, n (%)	Predominantly Receptive, Sometimes Active Music Therapy, n (%)	Predominantly Active, Sometimes Receptive Music Therapy, n (%)	Mixed Active and Receptive Music Therapy, n (%)
Patients, n (%)	32 (39)	24 (29)	2 (2)	6 (7)	19 (23)
Under 1 year, n (%) 1 to 3 years, n (%) 4 to 7 years, n (%) 8 to 10 years, n (%) 11 to 14 years, n (%) 15 to 18 years, n (%)	13 (41) 17 (53) 0 (0) 0 (0) 1 (3) 1 (3)	0 (0) 5 (21) 13 (54) 2 (8) 3 (13) 1 (4)	1 (50) 1 (50) 0 (0) 0 (0) 0 (0) 0 (0)	0 (0) 1 (17) 0 (0) 1 (17) 2 (33) 2 (33)	1 (5) 2 (11) 12 (63) 1 (5) 1 (5) 2 (11)

**Table 3:** Parents’ perceptions during inpatient music therapy care of their hospitalized child with different inpatient statuses of the parents.

		Mean (%) All Parents n = 83	Mean (%)		Mean Difference between Hospitalized and Non-Hospitalized Parents (95% CI)	*p*-Value (Hospitalized and Non-Hospitalized Parents)
			Hospitalized Parents n = 53	Non-Hospitalized Parents n = 28		
The music therapy support was a positive change and enrichment during the clinical stay.	Fully agree	96.4	96.2	96.4	−0.2 (−9.1–8.7)	0.964
	Rather agree	3.6	3.8	3.6	0.2 (−8.7–9.1)	0.964
	Rather disagree	0.0	0.0	0.0	-	-
	Disagree	0.0	0.0	0.0	-	-
	Don’t know/no answer	0.0	0.0	0.0	-	-
My daughter/son was able to relax well without having to exert herself/himself.	Fully agree	94.0	94.3	92.8	1.5 (−10.3–13.3)	0.803
	Rather agree	1.2	1.9	0.0	1.9 (−1.9–5.7)	0.322
	Rather disagree	1.2	0.0	3.5	−3.5 (−10.9–3.8)	0.326
	Disagree	0.0	0.0	0.0	-	-
	Don’t know/no answer	3.6	3.8	3.6	0.2 (−8.7–9.1)	0.964
My daughter/son was able to repress thoughts about her/his illness to some extent during the music therapy.	Fully agree	73.5	83.0	46.4	36.6 (14.6–58.6)	**0.002**
	Rather agree	7.2	5.6	10.7	−5.1 (−18.7–8.6)	0.459
	Rather disagree	0.0	0.0	0.0	-	-
	Disagree	0.0	0.0	0.0	-	-
	Don’t know/no answer	19.3	11.4	42.8	−31.6 (−52.8–(−10.3))	**0.005**
My daughter/son has gained more self-confidence in therapy.	Fully agree	60.2	66.0	46.4	19.6 (−3.7–42.9)	0.098
	Rather agree	4.8	5.7	3.5	2.2 (−7.5–11.7)	0.665
	Rather disagree	4.8	5.7	3.5	2.2 (−7.5–11.7)	0.665
	Disagree	0.0	0.0	0.0	-	-
	Don’t know/no answer	30.2	22.6	46.4	−23.8 (−46.4–(−1.2))	**0.039**
My daughter/son has developed an enjoyment of singing during the course of therapy.	Fully agree	59.1	62.2	50.0	12.2 (−11.3–35.8)	0.301
	Rather agree	4.8	7.6	.00	7.6 (0.2–14.9)	0.044
	Rather disagree	2.4	1.9	3.6	−1.7 (−9.8–6.5)	0.679
	Disagree	2.4	3.8	0.0	3.8 (−1.5–9.1)	0.159
	Don’t know/no answer	31.3	24.5	50.0	−25.5 (−48.2–(−2.7))	**0.029**
The therapist was empathetic and understanding.	Fully agree	96.4	98.2	92.8	5.4 (−5.5–16.0)	0.328
	Rather agree	3.3	1.8	7.1	−5.3 (−16.0–5.5)	0.328
	Rather disagree	0.0	0.0	0.0	-	-
	Disagree	0.0	0.0	0.0	-	-
	Don’t know/no answer	0.0	0.0	0.0	-	-
There have been things that have disrupted the sessions of music therapy.	Fully agree	0.0	0.0	0.0	-	-
	Rather agree	4.8	3.8	7.1	−3.3 (−14.7–8.0)	0.552
	Rather disagree	4.8	1.9	10.7	−8.8 (−21.5–3.9)	0.167
	Disagree	80.8	84.9	71.4	13.5 (−6.7–33.6)	0.185
	Don’t know/no answer	9.6	9.4	10.7	−1.3 (−15.7–13.2)	0.860
Overall, I am grateful for the music therapy for my child during the clinical stay.	Fully agree	96.4	96.2	96.4	−0.2 (−9.1–8.7)	0.964
	Rather agree	1.2	0.0	3.6	−3.6 (−10.9–3.8)	0.326
	Rather disagree	0.0	0.0	0.0	-	-
	Disagree	0.0	0.0	0.0	-	-
	Don’t know/no answer	2.4	3.8	0.0	3.8 (−1.5–9.1)	0.159

CI = confidence interval, Bold = significant.

## Data Availability

Original data will be made available to any qualified researcher upon request.
